# Comparison of the efficacy of anti-diabetic medications as add-on to metformin in type 2 diabetes mellitus from a real-world database

**DOI:** 10.1186/s40360-023-00716-4

**Published:** 2023-12-09

**Authors:** Ryosuke Ono, Chika Ogami, Chihiro Hasegawa, Hideto To, Yoshiaki Matsumoto, Yasuhiro Tsuji

**Affiliations:** 1 Laboratory of Clinical Pharmacometrics, School of Pharmacy, Nihon University, 7-7-1 Narashinodai, Funabashi, Chiba, 274-8555 Japan; 2 Clinical Pharmacology and Bioanalytics, Pfizer R&D Japan, 3-22-7 Yoyogi, Shibuya-Ku, Tokyo, 151-8589 Japan; 3 Department of Medical Pharmaceutics, Graduate School of Medical and Pharmaceutical Sciences for Research, University of Toyama, 2630 Sugitani, Toyama City, 930-0194 Japan

**Keywords:** HbA1c, Monotherapy, Interaction of metformin, Hypoglycemic agents, Dipeptidyl peptidase-4 inhibitor, Sodium-glucose co-transporter-2 inhibitor

## Abstract

**Background:**

Metformin is recommended as a first-line drug in the guidelines of the treatment for type 2 diabetes mellitus. However, high-quality evidence from clinical trials directly comparing the degree of hypoglycemic effect of combination therapy of metformin and a hypoglycemic agent with a different mechanism of action with that of monotherapy of a hypoglycemic drug is lacking. We aimed to examine whether combination therapy of hypoglycemic agents with metformin showed antagonism, addition, or synergism compared to monotherapy with hypoglycemic agents other than metformin regarding hemoglobin A_1c_ levels.

**Methods:**

This retrospective cohort study used a medical information database in Japan. Non-insulin anti-hyperglycemic agents with different mechanisms of action were classified into eight drug classes. A monotherapy cohort and a combination therapy added to the metformin cohort were defined. The change in hemoglobin A_1c_ levels was evaluated to compare the treatment effect between the cohorts.

**Results:**

A total of 13,359 patients with type 2 diabetes mellitus in the monotherapy cohort and 1,064 in the metformin combination therapy cohort were identified. A comparison of the change from baseline HbA1c level by drug class between the two cohorts showed a similar trend. Among those treated with dipeptidyl peptidase-4 inhibitor and sodium-glucose co-transporter-2 inhibitor, no clinically significant difference was observed between the two cohorts (0.00% and -0.07% for unadjusted, 0.15% and -0.03% for propensity score matching-adjusted, and 0.09% and -0.01% for inverse probability treatment weighting-adjusted analysis).

**Conclusions:**

According to the results of this study, the effect of dipeptidyl peptidase-4 inhibitor or sodium-glucose co-transporter-2 inhibitor added to metformin seems to be additive with respect to the reduction in hemoglobin A_1c_.

**Supplementary Information:**

The online version contains supplementary material available at 10.1186/s40360-023-00716-4.

## Background

The worldwide increase in the prevalence of type 2 diabetes mellitus (T2DM) is mostly attributed to an increase in the population of overweight and obese people [1]. An estimated 451 million people worldwide are affected by diabetes [2], and approximately 1 in 11 adults has diabetes, 90% of whom have T2DM [3]. Asia is a central region of the rapidly emerging T2DM pandemic, with China and India being the most affected countries [3]. According to the National Health and Nutrition Survey conducted by Ministry of Health, Labour and Welfare in 2019, approximately 15% of the adult population have diabetes (hemoglobin A_1c_ [HbA1c] ≥ 6.5% or under treatment for diabetes) and approximately 13% of the adult population are on the verge of developing diabetes (6.0% ≤ HbA1c < 6.5%) [4]. For every 1% decrease in HbA1c, the risk of microvascular complications decreases by 37% and that of diabetes-related death decreases by 21% [5].

While existing pharmacological options for the diabetes treatments may provide satisfactory glycemic control for some patients, there remains many patients who do not achieve the target HbA1c levels, suggesting the need for additional therapeutic options. In Western countries, metformin used to be recommended as a first-line drug in the guidelines of the treatment for T2DM [6], in recent years, the pharmacologic therapy should be guided by person-centered treatment factors, including comorbidities and treatment goals [7, 8]. Clinical practice guidelines for diabetes in Japan do not specify a first-line drug, and drug selection is left to the discretion of physicians in consideration of patient characteristics and pathological conditions [9]. Nevertheless, dipeptidyl peptidase-4 inhibitor (DPP-4i) is the most widely used drug for T2DM in Japan, followed by metformin [10]. Additionally, the Japanese guidelines for the treatment of diabetes recommend combination therapy with hypoglycemic agents with different mechanisms of action if the target glycemic control level is not achieved by mono-therapy of first-line treatment [9]. Moreover, the early intervention with DPP-4i in combination with metformin provides more effective and long-lasting benefits than metformin monotherapy, which is the current standard of care [11]. The hypoglycemic effect of the addition of a hypoglycemic agent with a different mechanism of action to metformin has been reported by meta-analysis, and it has been reported that dual-drug combination therapy added to metformin is more effective than metformin monotherapy for lowering HbA1c levels among all drug classes [12, 13]. A clinical study comparing sulfonylurea (SU) monotherapy with combination therapy SU and metformin, reported that dual therapy with metformin had a higher hypoglycemic effect than SU monotherapy [14]. However, high-quality evidence from clinical trials directly comparing the degree of hypoglycemic effect of combination therapy of metformin and a hypoglycemic agent with a different mechanism of action with that of monotherapy of a hypoglycemic drug is lacking [15, 16]. Additionally, in previous meta-analyses, individual patient background data, such as factors affecting hypoglycemic action, have not been used for analysis.

With the recent proliferation of electronic medical records and administrative claims databases, the results of analyses of real-world data have become increasingly important in medical decision-making. Real-world database research is recognized as a powerful tool for understanding the impact of current practice on the clinical course and outcomes, including long-term glycemic control, incidence of microvascular and macrovascular disease, and mortality [10].

Therefore, we comprehensively evaluated the interaction for the efficacy of metformin with hypoglycemic agents that have different mechanisms of action using individual patient data obtained from the medical database for each drug class. We aimed to examine whether combination therapy of hypoglycemic agents with metformin showed antagonism, addition, or synergism compared to monotherapy with hypoglycemic agents other than metformin.

## Methods

### Study design and data source

This retrospective cohort study used data of patients with T2DM (International Classification of Disease 10th revision [ICD10]: E11-E14) purchased from medical institutions in Japan from April 2008 to May 2020 in the Medical Data Vision Corporation (MDV, Tokyo, Japan) database. We evaluated eight types of non-insulin anti-hyperglycemic agents (NAAs) with different mechanisms of action, which were classified into eight drug classes: metformin, DPP-4i, SU, thiazolidinedione (TZD), α-glucosidase inhibitor (α-GI), glinide, sodium-glucose co-transporter-2 inhibitor (SGLT2i), and glucagon like peptide-1 receptor (GLP-1Ra). Two cohorts were defined: a monotherapy cohort that was treated with a single agent other than metformin, and a combination therapy cohort that was treated with metformin followed by a hypoglycemic agent with a different mechanism of action was added after the treatment with metformin. The change from baseline in HbA1c was evaluated to compare the treatment effects between the two cohorts.

### Study population

**Fig. 1:**
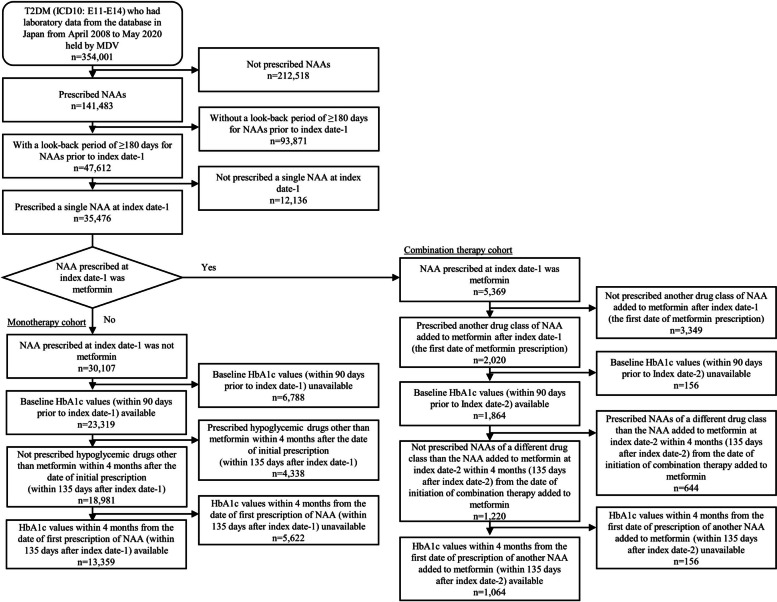
Flow chart describing the extraction of target patients of both cohorts for the analysis in this study

Data from patients prescribed NAAs were extracted, and the date of first prescription of NAAs was set as index date-1. According to a previous study, patients with a look-back period of at least 180 days prior to index date-1 were selected to target hypoglycemic drug-naïve patients with T2DM [17]. Patients with a single NAA prescribed on index date-1 were selected. If the NAA prescribed on index date-1 was a drug other than metformin, the patient was assigned to the monotherapy cohort and classified into a drug class according to the drug. The combination therapy cohort was defined as patients who were prescribed metformin at index date-1, followed by another NAA was added to metformin. For the combination therapy cohort, index date-2 was defined as the first prescription date of another NAA. Inclusion and exclusion criteria are shown in Fig. 1.

### Outcome measures

The primary endpoint was the change from baseline in HbA1c at 4 months (75 to 135 days) after index date-1 for the monotherapy cohort, and at 4 months (75 to 135 days) after index date-2 for the combination therapy cohort. We defined 0.4% as a clinically meaningful difference, as it is the generally accepted non-inferior margin value for changes in HbA1c among those with diabetes mellitus [18]. Furthermore, we defined a difference of the change from baseline in HbA1c from the combination therapy cohort relative to the monotherapy cohort within ± 0.4% as additive, ≥  + 0.4% as antagonistic, and ≤ -0.4% as synergistic.

### Statistical analysis

#### Index date

Index date for each cohort was defined index date-1 for the monotherapy cohort and index date-2 for the combination therapy added to metformin cohort.

#### Scatter plot of change from baseline in HbA1c by 4 months

The change from baseline in HbA1c by 4 months (135 days) from the index date of each cohort was plotted by cohort and drug class.

#### Mixed Model Repeated Measures (MMRM)

For primary endpoint analysis, the difference between the two cohorts by drug class was evaluated by the change from baseline in HbA1c from the index date to 4 months using MMRM. The change from baseline at 1 month (1–44 days), 2 months (45–74 days), and 4 months (75–135 days) as time-point were included in MMRM. If the same patient had multiple HbA1c values within the same time-point, the HbA1c value on the latest test date within that time-point was used. The model included cohort, time-point, interaction between cohort and time-point, baseline HbA1c value, age category (≤ 64, 65–74, ≥ 75 years), sex, NAAs and dose amount as fixed effects, and the correlation structure of the first-order autoregressive model (AR(1)) was used within patient correlations from the designated time-point to 4 months. Missing at Random was assumed for the missing measurement mechanism, and analysis by MMRM was performed using only the measured data.

In addition to the analysis using the analysis population (hereafter referred to as “unadjusted analysis”), two analyses using propensity score, which is an index that aggregates information on multiple confounding factors into a single value, were also performed.

#### Unadjusted analysis

The change from baseline in HbA1c to 4 months after the index date was evaluated using MMRM (refer to “Mixed Model Repeated Measures (MMRM)” section for the model) in the analysis population with unadjusted propensity scores (unadjusted analysis).

#### Propensity Score Matching (PSM) analysis

We performed 1:1 matching without replacement (nearest-neighbor method using calipers of width equal to 0.2 of the standard deviation of the logit of the propensity score). Baseline HbA1c value, age category, sex, NAAs and dose amount, complications profile (hypertension, ischemic heart disease, myocardial infarction, heart failure, stroke, renal impairment and diabetic foot) [17], contraindications to metformin [renal impairment, severe hepatic impairment [19], heart failure, myocardial infarction and type 1 diabetes mellitus (T1DM)] and other indications except T2DM of each drug (T1DM) were used as covariates in the propensity score model. The change from baseline in HbA1c to 4 months after the index date was evaluated using MMRM (refer to “Mixed Model Repeated Measures (MMRM)” section for the model) for matched patients.

#### Inverse Probability Treatment Weighting (IPTW) analysis

IPTW was performed on the analysis population using MMRM (refer to “Mixed Model Repeated Measures (MMRM)” section for the model) of the change from baseline in HbA1c to 4 months after the index date. The inverse of the propensity score estimated from PSM was used to estimate the weight.

This analysis was performed using Python version 3.8.5 with Anaconda 3 version 4.9.2 (Anaconda, Inc.), R version 3.5.1, and SAS version 9.4 (SAS Institute, Cary, NC, USA).

#### Sensitivity analysis for those with ≥ 90 days between index date-1 and index date-2 in combination therapy cohort

For the combination therapy cohort, a subset of patients was defined as those with ≥ 90 days between index date-1 and index date-2. The change from baseline in HbA1c to 4 months after the index date was evaluated using MMRM (refer to “Mixed Model Repeated Measures (MMRM)” section for the model) in the subset.

## Results

### Study population

Patients who met the eligibility criteria for this study were identified and 13,359 patients were included in the monotherapy cohort and 1,064 in the combination cohort (Fig. 1).

### Descriptive statistics for demographics and characteristics

The demographic and disease characteristics at the index date for each cohort are summarized by drug class in Table 1. DPP4i was the most prescribed drug class in both cohorts. In the combination therapy cohort, SGLT2i the next most common drug class. The baseline HbA1c values in the monotherapy cohort were highest for those prescribed GLP-1Ra. The number of patients was limited in the SU, TZD, α-GI, glinide and GLP-1Ra drug classes in the combination therapy cohort. Baseline HbA1c levels tended to be higher in the combination therapy cohort than in the monotherapy cohort. Age at the index date was lower in the combination therapy cohort than the monotherapy cohort. The incidence of ischemic heart disease, heart failure, and renal impairment tended to be lower in the combination therapy cohort than the monotherapy cohort.

**Table 1:** Baseline characteristics in each cohort

Cohort	Monotherapy cohort at Index date-1 (*N* = 13,359)								Combination therapy added on metformin cohort at Index date-2 (*N* = 1,064)							
Drug class	DPP-4i	SU	TZD	α-GI	Glinide	SGLT2i	GLP-1 Ra	Total Mono	DPP-4i	SU	TZD	α-GI	Glinide	SGLT2i	GLP-1 Ra	Total Comb
n	10,111	602	219	956	301	872	298	13,359	688	51	32	28	16	220	29	1,064
% Female	38.7	38.2	42.9	40.4	33.9	36.4	43.0	38.7	41.1	29.4	53.1	28.6	37.5	35.9	72.4	40.3
HbA1c (%)	7.4 (1.3)	7.7 (1.7)	7.3 (1.6)	7.2 (1.3)	7.3 (1.7)	7.6 (1.4)	8.8 (2.3)	7.5 (1.4)	8.2 (1.6)	9.2 (1.8)	8.6 (1.9)	7.9 (1.6)	7.3 (1.0)	8.4 (1.9)	8.3 (1.9)	8.3 (1.7)
HbA1c (mmol/mol)	58 (15)	61 (18)	56 (18)	55 (15)	57 (19)	60 (15)	73 (25)	58 (15)	66 (17)	77 (20)	70 (20)	63 (18)	56 (11)	69 (20)	68 (20)	67 (18)
Age (years)	69.3 (11.8)	71.1 (11.4)	67.0 (12.7)	68.1 (12.8)	70.3 (11.6)	60.4 (13.7)	61.4 (15.2)	68.6 (12.3)	60.6 (13.0)	59.4 (13.2)	56.7 (11.9)	55.6 (17.1)	61.1 (16.4)	51.9 (12.5)	48.5 (12.5)	58.2 (13.5)
≤ 64	3,030 (30.0)	144 (23.9)	85 (38.8)	318 (33.3)	78 (25.9)	495 (56.8)	163 (54.7)	4,313 (32.3)	381 (55.4)	30 (58.8)	21 (65.6)	18 (64.3)	8 (50.0)	178 (80.9)	26 (89.7)	662 (62.2)
65–74	3,436 (34.0)	205 (34.1)	68 (31.1)	316 (33.1)	108 (35.9)	248 (28.4)	70 (23.5)	4,451 (33.3)	226 (32.8)	14 (27.5)	11 (34.4)	7 (25.0)	3 (18.8)	39 (17.7)	3 (10.3)	303 (28.5)
≥ 75	3,645 (36.0)	253 (42.0)	66 (30.1)	322 (33.7)	115 (38.2)	129 (14.8)	65 (21.8)	4,595 (34.4)	81 (11.8)	7 (13.7)	0	3 (10.7)	5 (31.3)	3 (1.4)	0	99 (9.3)
Comorbidities (%)																
Hypertension	6770 (67.0)	395 (65.6)	151 (68.9)	595 (62.2)	198 (65.8)	580 (66.5)	176 (59.1)	8865 (66.4)	416 (60.5)	29 (56.9)	18 (56.3)	15 (53.6)	10 (62.5)	113 (51.4)	16 (55.2)	617 (58.0)
Ischaemic heart disease	2703 (26.7)	176 (29.2)	47 (21.5)	265 (27.7)	80 (26.6)	271 (31.1)	55 (18.5)	3597 (26.9)	121 (17.6)	9 (17.6)	2 (6.3)	6 (21.4)	1 (6.3)	25 (11.4)	1 (3.4)	165 (15.5)
Myocardial infarction	716 (7.1)	39 (6.5)	8 (3.7)	55 (5.8)	17 (5.6)	93 (10.7)	16 (5.4)	944 (7.1)	30 (4.4)	4 (7.8)	2 (6.3)	2 (7.1)	0	5 (2.3)	0	43 (4.0)
Heart failure	2327 (23.0)	113 (18.8)	27 (12.3)	172 (18.0)	82 (27.2)	244 (28.0)	60 (20.1)	3025 (22.6)	73 (10.6)	6 (11.8)	3 (9.4)	2 (7.1)	1 (6.3)	14 (6.4)	3 (10.3)	102 (9.6)
Stroke	2115 (20.9)	120 (19.9)	49 (22.4)	165 (17.3)	48 (15.9)	118 (13.5)	52 (17.4)	2667 (20.0)	111 (16.1)	7 (13.7)	4 (12.5)	5 (17.9)	3 (18.8)	14 (6.4)	7 (24.1)	151 (14.2)
Renal impairment	1916 (18.9)	59 (9.8)	30 (13.7)	194 (20.3)	73 (24.3)	107 (12.3)	81 (27.2)	2460 (18.4)	57 (8.3)	5 (9.8)	6 (18.8)	4 (14.3)	1 (6.3)	39 (17.7)	4 (13.8)	116 (10.9)
Diabetic foot	269 (2.7)	19 (3.2)	6 (2.7)	27 (2.8)	12 (4.0)	17 (1.9)	13 (4.4)	363 (2.7)	10 (1.5)	0	1 (3.1)	1 (3.6)	0	1 (0.5)	1 (3.4)	14 (1.3)
Hepatic impairment - Severe	530 (5.2)	42 (7.0)	14 (6.4)	49 (5.1)	15 (5.0)	21 (2.4)	10 (3.4)	681 (5.1)	21 (3.1)	2 (3.9)	3 (9.4)	1 (3.6)	1 (6.3)	2 (0.9)	2 (6.9)	32 (3.0)
Lactic acidosis	1 (0.0)	0	0	1 (0.1)	0	0	0	2 (0.0)	0	0	0	0	0	0	0	0
T1DM	96 (0.9)	6 (1.0)	1 (0.5)	80 (8.4)	5 (1.7)	61 (7.0)	3 (1.0)	252 (1.9)	5 (0.7)	1 (2.0)	0	1 (3.6)	0	4 (1.8)	1 (3.4)	12 (1.1)

Data are presented as mean (standard deviation), or n (%)

*Abbreviations*: *Mono* Monotherapy therapy, *Comb* Combination therapy

### Descriptive statistics for each drug and dose amount

The NAAs and dose amount of the drug classes are summarized in Table S1. No significant bias was observed in the proportion of NAA or dose amount between the cohorts. Because the entry rules for the dose amount of GLP-1Ra, which are injectable agents, differ depending on the hospital, it was not possible to identify the dose amount from the database.

### Scatter plot of change from baseline in HbA1c by 4 months

**Fig. 2:**
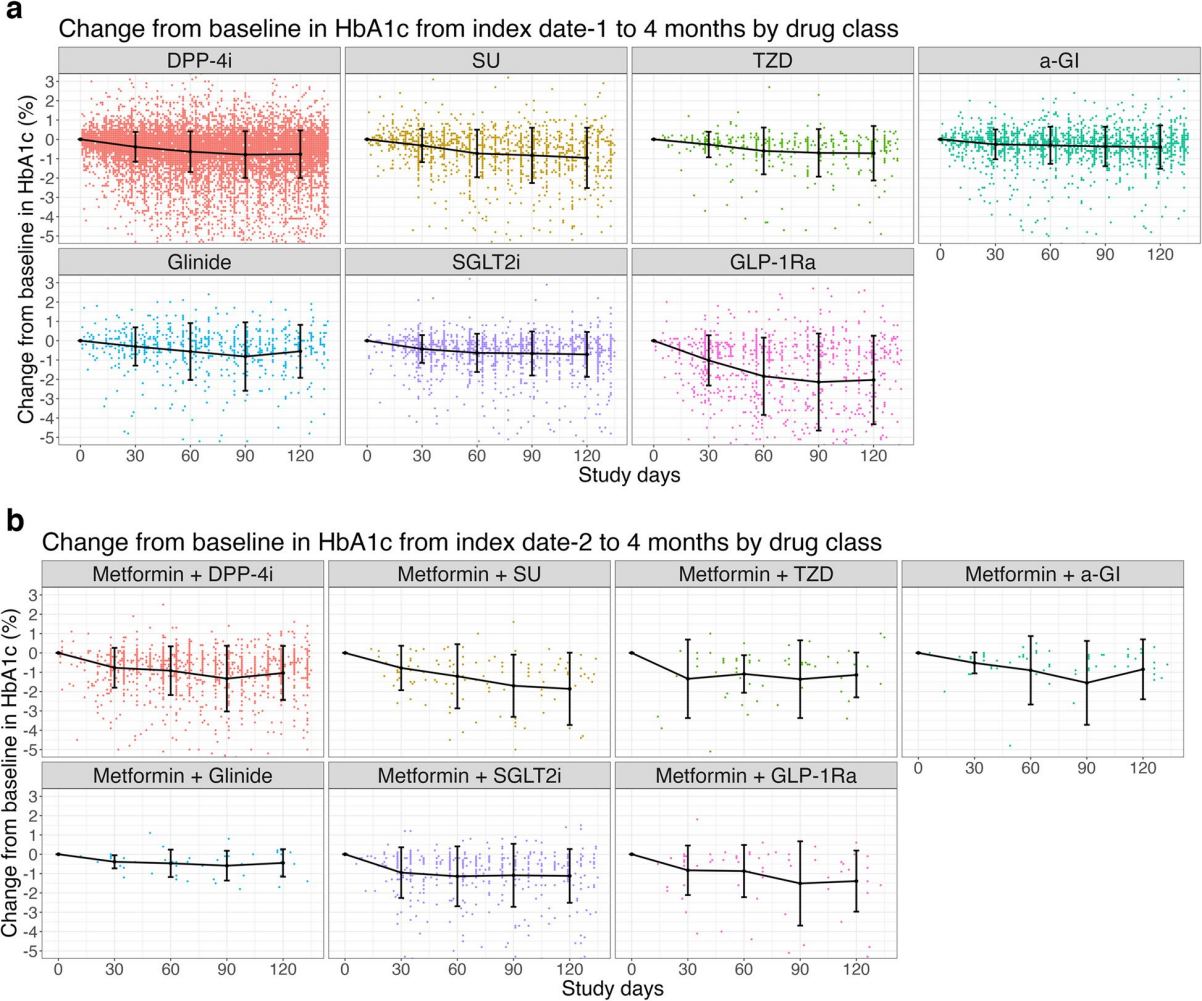
Plot of change from baseline HbA1c for monotherapy cohort (**a**) and combination therapy cohort (**b**)

The individual values and mean ± standard deviation of the change from baseline in HbA1c to 4 months for each cohort were plotted for each drug class (Fig. 2). In both cohorts, HbA1c values decreased over time after the first prescription date of NAAs, as well as the date of concomitant prescription with metformin and stabilized after 3–4 months. In the monotherapy cohort, a remarkable hypoglycemic effect was observed especially with GLP-1Ra. The trend of change from baseline in HbA1c value according to drug class was generally similar between the two cohorts.

### Unadjusted analysis

**Fig. 3:**
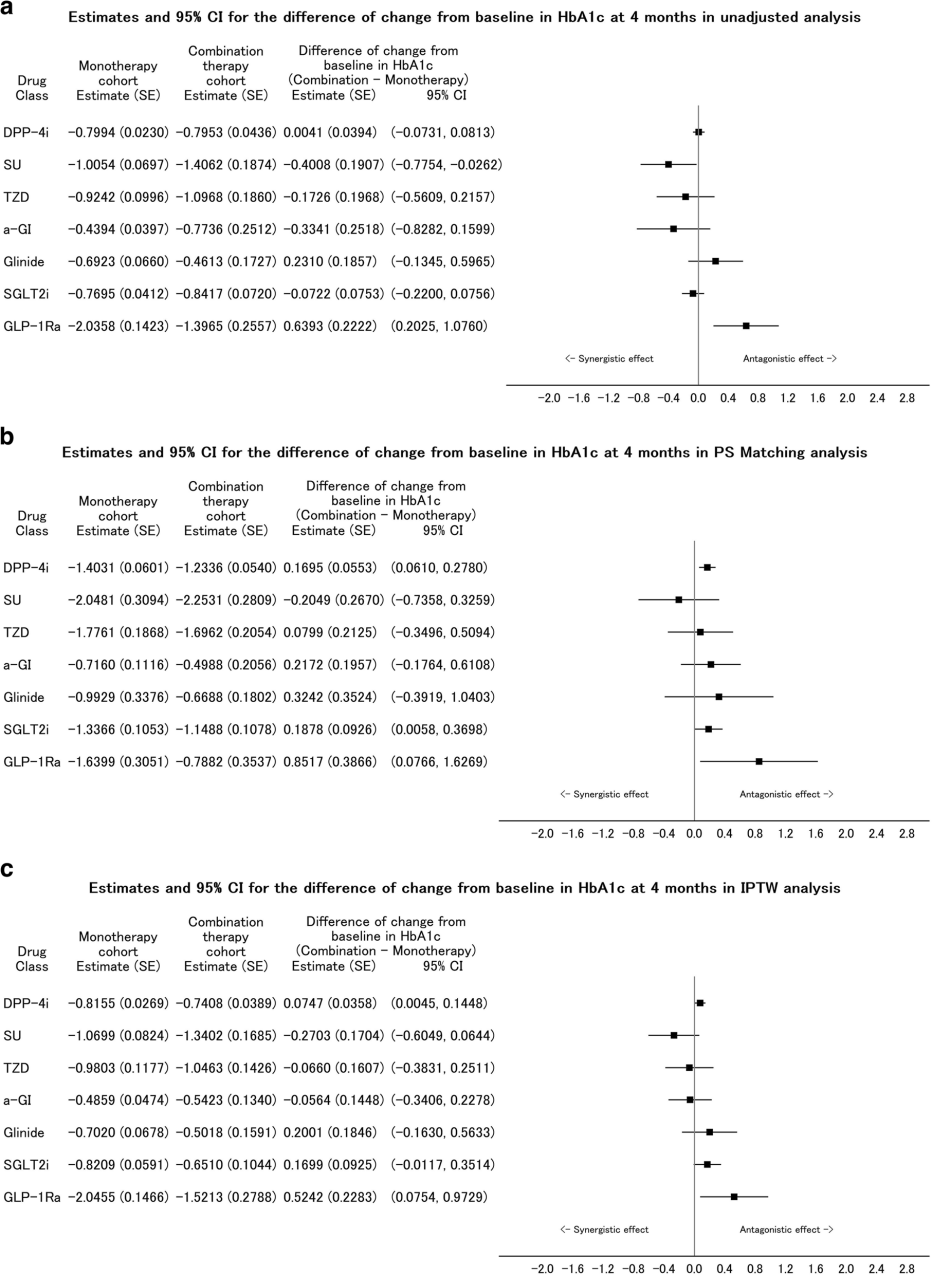
Plot of MMRM for difference of change from baseline HbA1c at four months between cohorts with unadjusted analysis (**a**), PSM-adjusted analysis (**b**), and IPTW-adjusted analysis (**c**)

No clinically meaningful difference was observed between the two cohorts except for SU and GLP-1Ra (Fig. 3a). The results of the parameter estimates by MMRM are shown in Table S2.

### PSM analysis

No clinically meaningful difference was observed between the two cohorts for except for GLP-1Ra (Fig. 3b).

### IPTW analysis

In IPTW, no clinically meaningful difference was observed between the two cohorts except for GLP-1Ra (Fig. 3c).

### Sensitivity analysis for those with ≥ 90 days between index date-1 and index date-2 in combination therapy cohort

Results of the sensitivity analysis are shown in Fig. S1. The number of patients in the combination therapy cohort was further decreased (Table S3). The confidence interval of the difference between the two cohorts in the change from baseline in HbA1c was wider in the subset compared to the entire analysis population. The change from baseline in HbA1c of the combination therapy cohort in the subset tended to be lower than that in the entire analysis population.

## Discussion

The MDV has approximately 29.8 million inpatient and outpatient records from approximately 400 hospitals that have accumulated since April 2008, covering approximately 22% of the Japanese population [10]. The database has several advantages. First, it contains a wealth of data on the elderly population. Second, it is more reliable than receipt data because it is collected to review the quality of medical care. Third, although data is limited to approximately 20% of the patients included in the database, information on common laboratory results is available.

At index date for each cohort, those prescribed GLP-1Ra have the highest baseline HbA1c values. This indicates that GLP-1Ra was prescribed as the first NAA in patients with significantly poor glycemic control. Moreover, baseline HbA1c levels tended to be higher in the combination therapy cohort than the monotherapy cohort, suggesting that patients with poor glycemic control were often shifted to treatment with combination therapy with other classes of drugs. The lower age of the combination therapy cohort compared to the monotherapy cohort indicated that metformin should be used carefully in elderly patients [9]. The lower incidence of ischemic heart disease, heart failure, and renal dysfunction in the combination therapy cohort compared to the monotherapy cohort indicated that these comorbidities are relevant to contraindications of metformin. For the factors that may be associated with the change from baseline in HbA1c values, the potential confounding effect on the estimation of HbA1c changes was minimized (adjusted) as covariates of MMRM and/or using propensity score. Besides, the immortal time bias seems to be decreased because the index date for the combination therapy cohort was defined as index date-2. On the other hand, the selection bias to define the index date for the combination therapy cohort as index date-2 should be considered; however, the selection bias seems to be decreased by conducting PSM- and IPTW-adjusted analyses.

The direct linkage between drug and indication is unavailable based on the specification of the MDV database. Out of all NAAs in this study, a few NAAs have other indications except T2DM, such as, dapagliflozin propylene glycolate hydrate has other indications for chronic heart failure (approved date in Japan: 27 November 2020), chronic kidney disease (CKD, approved date in Japan: 25 August 2021) and T1DM (approved date in Japan: 26 March 2019), empagliflozin has another indication for chronic heart failure (approved date in Japan: 25 November 2021), and ipragliflozin L-proline has another indication for T1DM (approved date in Japan: 21 December 2018). Considering the observation period of this study is from April 2008 to May 2020, dapagliflozin propylene glycolate hydrate and empagliflozin for the treatment of chronic heart failure, and dapagliflozin propylene glycolate hydrate for the treatment of CKD have not been applied during this study period. And it seems that the impact of usage for T1DM (dapagliflozin propylene glycolate hydrate and ipragliflozin L-proline) was minimal during this study period based on the tiny rate of T1DM. However, other indications except T2DM defined as T1DM for dapagliflozin propylene glycolate hydrate and ipragliflozin L-proline, and the covariate has been included in the PS model.

Due to the high prescription rates of DPP4i and SGLT2i in Japan [20], these drugs were considered to have sufficient data in both cohorts for analysis. However, for SU, TZD, α-GI, glinide, and GLP-1Ra, the interpretation of the hypoglycemic effects was limited because the number of patients in the combination therapy cohort was too small to be analyzed. In addition, because only the subcutaneous injection product was marketed for GLP-1Ra during the survey period, the number of patients suitable for analysis may have been limited.

Comparison of the prescribing proportion based on each NAA and dose amount within each drug class between both cohorts showed no major bias, indicating that no substantial difference was observed in the prescription of other drug classes in combination therapy with metformin compared to monotherapy. Therefore, the difference in drug prescription between the two cohorts did not substantially affect the change in HbA1c in either cohort.

Regarding the bodyweight, the weight loss is known to reverse the underlying metabolic abnormalities of type 2 diabetes and improve glucose control. In an analysis of randomized controlled trials after 2 years of follow-up, the loss of 15% of bodyweight can result in diabetes remission (ie, defined as HbA1c < 6.5% [< 48 mmol/mol]) in most patients with early type 2 diabetes; however, the observed average weight loss is modest (ie, 1.4–1.9 kg) in adults with type 2 diabetes over 6–12 months of treatment [21]. On the other hand, a meta-analysis after 4 months of follow-up reported that body weight at the start of medication did not affect the improvement effect of HbA1c in any drug class [22]. Although the data on body size at the index date were not included in the database and hence were not used for this analysis, it is likely that bodyweight had little influence on the change from baseline in HbA1c, considering the observation period for this study was 4 months from the index date which is a short period for evaluating the effect on loss of bodyweight.

In both cohorts, the change from baseline in HbA1c values decreased over time after the first prescription date of NAAs and the date of concomitant prescription with metformin, and the change from baseline in HbA1c values stabilized after three to four months. The Guideline for Clinical Evaluation of Oral Hypoglycemic Agents in Japan requires at least 12 weeks as the study duration for HbA1c [23], and clinically valid changes in HbA1c were observed from the data in the medical information database. A comparison of the change from baseline in HbA1c between the two cohorts for each drug class showed that the change in HbA1c in the combination therapy cohort was similar to that in the monotherapy cohort. However, greater variability was observed in the combination therapy cohort than the monotherapy cohort.

The hypoglycemic effects were available for DPP-4i, SU, TZD, and α-GI at 12 weeks post-dose in previous studies and were comparable to the results the monotherapy cohort in of this study [24]. In addition, comparing the hypoglycemic effects of DPP-4i, SU, TZD, α-GI, glinide, and GLP-1Ra from previous studies were compared with those of the combination therapy cohort. However, results of our study varied slightly from those of previous studies [12, 15]. This discrepancy could be due to differences in patient backgrounds, as the previous study was a randomized controlled trial conducted in a population with limited patient background, whereas this study was based on real-world data obtained in a more general patient population.

MMRM was used to evaluate the change from baseline in HbA1c between the two cohorts. Because this was an observational study that was not randomized between the cohorts, various confounding factors may have prevented the accurate estimation of treatment effects. Therefore, in addition to the unadjusted analysis using the analysis population, PSM- and IPTW-adjusted analyses using the propensity score were also performed to control for confounding bias and ensure comparability between the cohorts. For DPP4i and SGLT2i, no clinically significant difference was observed between the two cohorts in any of the analyses (unadjusted, PSM-adjusted, and IPTW-adjusted). This suggests that DPP4i and SGLT2i provided additive effects in relation to HbA1c lowering when added to treatment with metformin. On the other hand, the interpretation of hypoglycemic effects was limited for SU, TZD, α-GI, glinide, and GLP-1Ra owing to the small number of patients in the combination therapy cohort. However, for TZD, α-GI, and glinide, no clinically substantial difference was observed between the two cohorts in either analysis.

For SU, the difference in the direction of synergism was observed only in the unadjusted analysis, however no clinically substantial difference was observed in the PSM- and IPTW-adjusted analyses adjusted by propensity score. In terms of GLP-1Ra, a notable difference was observed between both cohorts in either analysis; however, the mean absolute value of HbA1c at 4 months was 6.7% for the monotherapy cohort and 6.9% for the combination therapy cohort, showing favorable glycemic control (Table S4). For MMRM, the relationship between the change from baseline in HbA1c and the HbA1c baseline value was assumed to be linear. However, a further reduction in blood glucose levels was unlikely to occur near favorable HbA1c values, and the baseline HbA1c value was lower in the combination therapy cohort (8.3%) than in the monotherapy cohort (8.8%), which may have contributed to the marked difference in the change from baseline in HbA1c between both cohorts for the unadjusted analysis. Therefore, PSM- and IPTW-adjusted analyses using the propensity score were also performed to control for confounding bias and ensure comparability between the cohorts, however due to the small number of patients prescribed GLP-1Ra, each covariate including baseline in HbA1c may not have been completely balanced between both cohorts. A model analysis for such potential non-linearity in the change from baseline in HbA1c would be a subject for future studies.

In the sensitivity analysis of the subset of patients from the combination therapy cohort with ≥ 90 days between index date-1 and index date-2, the change from baseline in HbA1c values was evaluated. No clinically significant difference was observed for DPP4i and SGLT2i between the two cohorts in either analysis (unadjusted, PSM-adjusted, and IPTW-adjusted), similar to the results of the entire analysis population. This sensitivity analysis showed consistent results to those obtained in the entire analysis population, supporting that DPP4i and SGLT2i provided additive effects in relation to HbA1c lowering when added to treatment with metformin. However, the interpretation of the hypoglycemic effects of SU, TZD, α-GI, glinide, and GLP-1Ra is limited because of the extremely small number of patients in the subset in the combination therapy cohort.

By comparing the entire analysis population and with the sensitivity analysis using the subset, the difference between the two cohorts in the change from baseline in HbA1c values tended to be slightly positive (in the direction of antagonism) in the sensitivity analysis for most drugs. This result indicates that the hypoglycemic effect of metformin may be carried over if the duration between the first date of metformin prescription and the date of concomitant prescription of another NAA was < 90 days. However, the sensitivity analysis also showed no clinically significant difference between the cohorts for DPP4i and SGLT2i.

This study had several limitations. First, the medical data used in this study has the disadvantage that it is not traceable if the patients were transferred to other hospitals [10], and diagnoses and treatments performed at other hospitals were not recorded. Second, the small number of patients prescribed SU, TZD, α-GI, glinide, or GLP-1Ra drug classes in the combination therapy cohort limited the interpretation of the results. However, the number of patients prescribed DPP-4i or SGLT2i drug classes in the combination therapy cohort was sufficient for interpretation. In addition, selection bias requires consideration because only a limited number of hospital laboratory values were included in the study. Although confounding adjustment was performed using propensity scores for factors considered related to changes in HbA1c, all necessary factors may not have been adjusted (e.g., duration of diabetes, history of hypoglycemia unawareness) due to the limited information available in the database.

## Conclusions

In conclusion, the degree of pharmacodynamic interaction of metformin with other hypoglycemic agents was comprehensively evaluated for each drug class using individual patient data obtained from the medical information database. According to the results of this study, the effect of DPP4i or SGLT2i added to metformin seems to be additive with respect to the reduction in HbA1c. Therefore, it is possible to infer what degree of hypoglycemic effect can be expected when DPP4i or SGLT2i is added to metformin, using public information such as clinical trial results of a drug that is classified as DPP4i or SGLT2i.

### Supplementary Information

**Additional file 1: Table S1.** Descriptive statistics for each drug and dose amount at index date in each cohort. **Table S2.** Summary of parameter estimates in MMRM with unadjusted analysis. **Table S3.** Baseline characteristics in each cohort: sensitivity analysis for ≥90 days between index date-1 and index date-2 in combination therapy cohort. **Table S4.** Descriptive summary of observed HbA1c value at 4 months with unadjusted population who had HbA1c value at 4 months. **Figure S1.** Plot of MMRM for difference of change from baseline in HbA1c at 4 months between cohorts with unadjusted analysis (a), PSM-adjusted analysis (b) and IPTW-adjusted analysis (c): Sensitivity analysis for patients with ≥90 days between index date-1 and index date-2 in combination therapy cohort.

## Data Availability

The datasets used and/or analysed during the current study are available from the corresponding author on reasonable request.

## Abbreviations

α-GI: α-Glucosidase inhibitor
DPP-4i: Dipeptidyl peptidase-4 inhibitor
GLP-1Ra: Glucagon like peptide-1 receptor
HbA1c: Hemoglobin A_1c_
ICD10: International Classification of Disease 10^th^ revision
IPTW: Inverse probability treatment weighting
MDV: Medical Data Vision Corporation
MMRM: Mixed model repeated measures
NAAs: Non-insulin anti-hyperglycemic agents
PSM: Propensity score matching
SGLT2i: Sodium-glucose co-transporter-2 inhibitor
SU: Sulfonylurea
T2DM: Type 2 diabetes mellitus
TZD: Thiazolidinedione
