# Supplementary material for: Comparison of the efficacy of anti-diabetic medications as add-on to metformin in type 2 diabetes mellitus from a real-world database
Source: BMC Pharmacol Toxicol. 2023 Dec 9;24:75. doi: 10.1186/s40360-023-00716-4 (PMC10709883; doi:10.1186/s40360-023-00716-4)
Supplement: Supplementary file 1 — Additional file 1: Table S1. Descriptive statistics for each drug and dose amount at index date in each cohort. Table S2. Summary of parameter estimates in MMRM with unadjusted analysis. Table S3. Baseline characteristics in each cohort: sensitivity analysis for ≥90 days between index date-1 and index date-2 in combination therapy cohort. Table S4. Descriptive summary of observed HbA1c value at 4 months with unadjusted population who had HbA1c value at 4 months. Figure S1. Plot of MMRM for difference of change from baseline in HbA1c at 4 months between cohorts with unadjusted analysis (a), PSM-adjusted analysis (b) and IPTW-adjusted analysis (c): Sensitivity analysis for patients with ≥90 days between index date-1 and index date-2 in combination therapy cohort. [file 40360_2023_716_MOESM1_ESM.docx]

# Supplemental Files

Table S1 Descriptive statistics for each drug and dose amount at index date in each cohort

|  |  |  | Monotherapy cohort |  | Combination therapy cohort |  |
| --- | --- | --- | --- | --- | --- | --- |
| Drug class | Drug name | Drug amount (mg) | n  (% in Drug class) |  | n  (% in Drug class) | Difference  % of drug  between cohorts |
| DPP-4i |  |  | N=10,111 |  | N=688 |  |
|  | Alogliptin Benzoate | 6.25 | 179 (1.8) |  | 21 (3.1) | 1.3 |
|  |  | 12.5 | 345 (3.4) |  | 38 (5.5) | 2.1 |
|  |  | 25 | 533 (5.3) |  | 24 (3.5) | -1.8 |
|  | Anagliptin | 100 | 78 (0.8) |  | 9 (1.3) | 0.5 |
|  | Linagliptin | 5 | 2511 (24.8) |  | 111 (16.1) | -8.7 |
|  | Omarigliptin | 12.5 | 10 (0.1) |  | 0 | -0.1 |
|  |  | 25 | 68 (0.7) |  | 3 (0.4) | -0.2 |
|  | Saxagliptin hydrate | 2.5 | 125 (1.2) |  | 18 (2.6) | 1.4 |
|  |  | 5 | 160 (1.6) |  | 26 (3.8) | 2.2 |
|  | Sitagliptin phosphate hydrate | 12.5 | 24 (0.2) |  | 0 | -0.2 |
|  |  | 25 | 808 (8.0) |  | 59 (8.6) | 0.6 |
|  |  | 50 | 2939 (29.1) |  | 239 (34.7) | 5.7 |
|  |  | 100 | 26 (0.3) |  | 8 (1.2) | 0.9 |
|  | Teneligliptin hydrobromide hydrate | 20 | 1156 (11.4) |  | 46 (6.7) | -4.7 |
|  | Trelagliptin succinate | 50 | 9 (0.1) |  | 0 | -0.1 |
|  |  | 100 | 87 (0.9) |  | 1 (0.1) | -0.7 |
|  | Vildagliptin | 50 | 1053 (10.4) |  | 85 (12.4) | 1.9 |
| SU |  |  | N=602 |  | N=51 |  |
|  | Glibenclamide | 1.25 | 28 (4.7) |  | 0 | -4.7 |
|  |  | 2.5 | 31 (5.1) |  | 0 | -5.1 |
|  | Gliclazide | 20 | 32 (5.3) |  | 9 (17.6) | 12.3 |
|  |  | 40 | 47 (7.8) |  | 1 (2.0) | -5.8 |
|  | Glimepiride | 0.5 | 94 (15.6) |  | 16 (31.4) | 15.8 |
|  |  | 1 | 359 (59.6) |  | 25 (49.0) | -10.6 |
|  |  | 3 | 11 (1.8) |  | 0 | -1.8 |
| TZD |  |  | N=602 |  | N=51 |  |
|  | Pioglitazone hydrochloride | 15 | 190 (86.8) |  | 24 (75.0) | -11.8 |
|  |  | 30 | 29 (13.2) |  | 8 (25.0) | 11.8 |
| α-GI |  |  | N=956 |  | N=28 |  |
|  | Acarbose | 50 | 60 (6.3) |  | 3 (10.7) | 4.4 |
|  |  | 100 | 22 (2.3) |  | 0 | -2.3 |
|  | Miglitol | 25 | 55 (5.8) |  | 4 (14.3) | 8.5 |
|  |  | 50 | 217 (22.7) |  | 5 (17.9) | -4.8 |
|  |  | 75 | 18 (1.9) |  | 0 | -1.9 |
|  | Voglibose | 0.2 | 377 (39.4) |  | 8 (28.6) | -10.9 |
|  |  | 0.3 | 207 (21.7) |  | 8 (28.6) | 6.9 |
| Glinide |  |  | N=301 |  | N=16 |  |
|  | Mitiglinide calcium hydrate | 5 | 44 (14.6) |  | 0 | -14.6 |
|  |  | 10 | 135 (44.9) |  | 9 (56.3) | 11.4 |
|  | Nateglinide | 30 | 15 (5.0) |  | 1 (6.3) | 1.3 |
|  |  | 90 | 28 (9.3) |  | 0 | -9.3 |
|  | Repaglinide | 0.25 | 51 (16.9) |  | 2 (12.5) | -4.4 |
|  |  | 0.5 | 28 (9.3) |  | 4 (25.0) | 15.7 |
| SGLT2i |  |  | N=872 |  | N=220 |  |
|  | Canagliflozin hydrate | 100 | 117 (13.4) |  | 41 (18.6) | 5.2 |
|  | Dapagliflozin propylene glycolate hydrate | 5 | 191 (21.9) |  | 41 (18.6) | -3.3 |
|  |  | 10 | 5 (0.6) |  | 0 | -0.6 |
|  | Empagliflozin | 10 | 307 (35.2) |  | 75 (34.1) | -1.1 |
|  |  | 25 | 4 (0.5) |  | 1 (0.5) | 0 |
|  | Ipragliflozin L-proline | 25 | 30 (3.4) |  | 12 (5.5) | 2 |
|  |  | 50 | 88 (10.1) |  | 21 (9.5) | -0.5 |
|  | Luseogliflozin hydrate | 2.5 | 55 (6.3) |  | 13 (5.9) | -0.4 |
|  |  | 5 | 1 (0.1) |  | 1 (0.5) | 0.3 |
|  | Tofogliflozin hydrate | 20 | 74 (8.5) |  | 15 (6.8) | -1.7 |
| GLP-1Ra |  |  | N=298 |  | N=29 |  |
|  | Dulaglutide (genetical recombination) | NA | 112 (37.6) |  | 9 (31.0) | -6.5 |
|  | Exenatide | NA | 3 (1.0) |  | 0 | -1 |
|  | Liraglutide (genetical recombination) | NA | 174 (58.4) |  | 20 (69.0) | 10.6 |
|  | Lixisenatide | NA | 9 (3.0) |  | 0 | -3 |

Table S2 Summary of parameter estimates in MMRM with unadjusted analysis

| Drug class | Effect | Estimate (SE) |
| --- | --- | --- |
| DPP-4i | Intercept | 3.0930 (0.0703) |
|  | Treatment |  |
|  | Combination therapy | 0.0041 (0.0394) |
|  | Monotherapy | 0 |
|  | Baseline value of HbA1c | -0.5142 (0.0095) |
|  | Sex |  |
|  | Male | -0.0216 (0.0124) |
|  | Female | 0 |
|  | Age |  |
|  | ≤64 | -0.0391 (0.0157) |
|  | 65-74 | -0.0388 (0.0137) |
|  | ≥75 | 0 |
|  | Drug Label |  |
|  | Alogliptin Benzoate 12.5 mg | 0.0188 (0.0372) |
|  | Alogliptin Benzoate 25 mg | 0.0071 (0.0307) |
|  | Alogliptin Benzoate 6.25 mg | -0.0057 (0.0447) |
|  | Anagliptin 100 mg | -0.0424 (0.0721) |
|  | Linagliptin 5 mg | 0.0013 (0.0229) |
|  | Omarigliptin 12.5 mg | 0.0111 (0.2001) |
|  | Omarigliptin 25 mg | 0.0689 (0.0876) |
|  | Saxagliptin hydrate 2.5 mg | 0.0296 (0.0538) |
|  | Saxagliptin hydrate 5 mg | 0.1198 (0.0460) |
|  | Sitagliptin phosphate hydrate 12.5 mg | 0.3416 (0.1698) |
|  | Sitagliptin phosphate hydrate 25 mg | 0.0923 (0.0271) |
|  | Sitagliptin phosphate hydrate 50 mg | 0.0083 (0.0223) |
|  | Sitagliptin phosphate hydrate 100 mg | -0.0733 (0.1510) |
|  | Teneligliptin hydrobromide hydrate 20 mg | -0.0010 (0.0261) |
|  | Trelagliptin succinate 50 mg | 0.0145 (0.1569) |
|  | Trelagliptin succinate 100 mg | 0.0207 (0.0539) |
|  | Vildagliptin 50 mg | 0 |
| SU | Intercept | 2.5940 (0.3319) |
|  | Treatment |  |
|  | Combination therapy | -0.4008 (0.1907) |
|  | Monotherapy | 0 |
|  | Baseline value of HbA1c | -0.4705 (0.0338) |
|  | Sex |  |
|  | Male | 0.0385 (0.0684) |
|  | Female | 0 |
|  | Age |  |
|  | ≤64 | 0.1512 (0.0847) |
|  | 65-74 | -0.0588 (0.0713) |
|  | ≥75 | 0 |
|  | Drug Label |  |
|  | Glibenclamide 1.25 mg | 0.2944 (0.3166) |
|  | Glibenclamide 2.5 mg | 0.1243 (0.2732) |
|  | Gliclazide 20 mg | -0.1297 (0.2721) |
|  | Gliclazide 40 mg | 0.0230 (0.2769) |
|  | Glimepiride 0.5 mg | 0.1943 (0.2645) |
|  | Glimepiride 1 mg | 0.1100 (0.2566) |
|  | Glimepiride 3 mg | 0 |
| TZD | Intercept | 2.7264 (0.3645) |
|  | Treatment |  |
|  | Combination therapy | -0.1726 (0.1968) |
|  | Monotherapy | 0 |
|  | Baseline value of HbA1c | -0.5066 (0.0506) |
|  | Sex |  |
|  | Male | 0.1255 (0.0777) |
|  | Female | 0 |
|  | Age |  |
|  | ≤64 | 0.2048 (0.0915) |
|  | 65-74 | -0.0173 (0.0888) |
|  | ≥75 | 0 |
|  | Drug Label |  |
|  | Pioglitazone hydrochloride 15 mg | 0.1541 (0.1420) |
|  | Pioglitazone hydrochloride 30 mg | 0 |
| α-GI | Intercept | 2.5395 (0.2752) |
|  | Treatment |  |
|  | Combination therapy | -0.3341 (0.2518) |
|  | Monotherapy | 0 |
|  | Baseline value of HbA1c | -0.4063 (0.0398) |
|  | Sex |  |
|  | Male | -0.0582 (0.0447) |
|  | Female | 0 |
|  | Age |  |
|  | ≤64 | -0.0108 (0.0502) |
|  | 65-74 | -0.0649 (0.0483) |
|  | ≥75 | 0 |
|  | Drug Label |  |
|  | Acarbose 100 mg | -0.1132 (0.1385) |
|  | Acarbose 50 mg | -0.1051 (0.0728) |
|  | Miglitol 25 mg | 0.2160 (0.1032) |
|  | Miglitol 50 mg | 0.2103 (0.0726) |
|  | Miglitol 75 mg | 0.1310 (0.1492) |
|  | Voglibose 0.2 mg | -0.0170 (0.0596) |
|  | Voglibose 0.3 mg | 0 |
| Glinide | Intercept | 3.4965 (0.3131) |
|  | Treatment |  |
|  | Combination therapy | 0.2310 (0.1857) |
|  | Monotherapy | 0 |
|  | Baseline value of HbA1c | -0.5747 (0.0372) |
|  | Sex |  |
|  | Male | 0.0085 (0.0815) |
|  | Female | 0 |
|  | Age |  |
|  | ≤64 | 0.0098 (0.1052) |
|  | 65-74 | -0.0130 (0.0895) |
|  | ≥75 | 0 |
|  | Drug Label |  |
|  | Mitiglinide calcium hydrate 5 mg | 0.1665 (0.2030) |
|  | Mitiglinide calcium hydrate 10 mg | -0.0378 (0.1735) |
|  | Nateglinide 30 mg | 0.3063 (0.2087) |
|  | Nateglinide 90 mg | 0.0283 (0.2179) |
|  | Repaglinide 0.25 mg | -0.1297 (0.1955) |
|  | Repaglinide 0.5 mg | 0 |
| SGLT2i | Intercept | 2.9751 (0.2269) |
|  | Treatment |  |
|  | Combination therapy | -0.0722 (0.0753) |
|  | Monotherapy | 0 |
|  | Baseline value of HbA1c | -0.5124 (0.0297) |
|  | Sex |  |
|  | Male | -0.0833 (0.0416) |
|  | Female | 0 |
|  | Age |  |
|  | ≤64 | -0.0089 (0.0502) |
|  | 65-74 | 0.0324 (0.0531) |
|  | ≥75 | 0 |
|  | Drug Label |  |
|  | Canagliflozin hydrate 100 mg | 0.1745 (0.0867) |
|  | Dapagliflozin propylene glycolate hydrate 5 mg | 0.4478 (0.0889) |
|  | Dapagliflozin propylene glycolate hydrate 10 mg | 0.0116 (0.1475) |
|  | Empagliflozin 10 mg | 0.2492 (0.0858) |
|  | Empagliflozin 25 mg | 0.3293 (0.1055) |
|  | Ipragliflozin L-proline 25 mg | 0.5301 (0.1086) |
|  | Ipragliflozin L-proline 50 mg | 0.5037 (0.1188) |
|  | Luseogliflozin hydrate 2.5 mg | 0.3083 (0.0961) |
|  | Luseogliflozin hydrate 5 mg | 0.5784 (0.1637) |
|  | Tofogliflozin hydrate 20 mg | 0 |
| GLP-1Ra | Intercept | 4.7407 (0.4081) |
|  | Treatment |  |
|  | Combination therapy | 0.6393 (0.2222) |
|  | Monotherapy | 0 |
|  | Baseline value of HbA1c | -0.6733 (0.0251) |
|  | Sex |  |
|  | Male | -0.0576 (0.1038) |
|  | Female | 0 |
|  | Age |  |
|  | ≤64 | -0.2302 (0.1442) |
|  | 65-74 | -0.1950 (0.1550) |
|  | ≥75 | 0 |
|  | Drug Label |  |
|  | Dulaglutide (genetical recombination) | -0.6696 (0.3830) |
|  | Exenatide | -0.9650 (0.4875) |
|  | Liraglutide (genetical recombination) | -0.7528 (0.3797) |
|  | Lixisenatid | 0 |

MMRM, mixed model repeated measures

Table S3 Baseline characteristics in each cohort: sensitivity analysis for ≥90 days between index date-1 and index date-2 in combination therapy cohort

| Cohort |  | Monotherapy cohort at Index date-1 (N=13,359) | | | | | | | |  | Combination therapy added on metformin cohort at Index date-2 (N=725) | | | | | | | |
| --- | --- | --- | --- | --- | --- | --- | --- | --- | --- | --- | --- | --- | --- | --- | --- | --- | --- | --- |
| Drug class |  | DPP-4i | SU | TZD | α-GI | Glinide | SGLT2i | GLP-1 Ra | Total Mono |  | DPP-4i | SU | TZD | α-GI | Glinide | SGLT2i | GLP-1 Ra | Total Comb |
| n |  | 10,111 | 602 | 219 | 956 | 301 | 872 | 298 | 13,359 |  | 478 | 23 | 18 | 15 | 9 | 165 | 17 | 725 |
| % Female |  | 38.7 | 38.2 | 42.9 | 40.4 | 33.9 | 36.4 | 43.0 | 38.7 |  | 42.5 | 30.4 | 50.0 | 13.3 | 44.4 | 37.0 | 70.6 | 41.1 |
| HbA1c (%) |  | 7.4 (1.3) | 7.7 (1.7) | 7.3 (1.6) | 7.2 (1.3) | 7.3 (1.7) | 7.6 (1.4) | 8.8 (2.3) | 7.5 (1.4) |  | 7.9 (1.2) | 8.5 (1.5) | 7.9 (1.0) | 7.3 (0.9) | 7.4 (1.2) | 8.0 (1.3) | 8.0 (1.6) | 7.9 (1.2) |
| HbA1c (mmol/mol) |  | 58 (15) | 61 (18) | 56 (18) | 55 (15) | 57 (19) | 60 (15) | 73 (25) | 58 (15) |  | 63 (13) | 69 (16) | 63 (10) | 57 (10) | 57 (13) | 64 (14) | 64 (17) | 63 (14) |
| Age (years) |  | 69.3 (11.8) | 71.1 (11.4) | 67.0 (12.7) | 68.1 (12.8) | 70.3 (11.6) | 60.4 (13.7) | 61.4 (15.2) | 68.6 (12.3) |  | 60.6 (13.0) | 59.0 (14.4) | 56.1 (10.7) | 52.5 (19.0) | 59.2 (18.3) | 51.7 (12.1) | 50.2 (9.8) | 58.0 (13.5) |
| ≤64 |  | 3,030 (30.0) | 144 (23.9) | 85 (38.8) | 318 (33.3) | 78 (25.9) | 495 (56.8) | 163 (54.7) | 4,313 (32.3) |  | 269 (56.3) | 13 (56.5) | 13 (72.2) | 12 (80.0) | 4 (44.4) | 137 (83.0) | 16 (94.1) | 464 (64.0) |
| 65-74 |  | 3,436 (34.0) | 205 (34.1) | 68 (31.1) | 316 (33.1) | 108 (35.9) | 248 (28.4) | 70 (23.5) | 4,451 (33.3) |  | 148 (31.0) | 7 (30.4) | 5 (27.8) | 2 (13.3) | 3 (33.3) | 27 (16.4) | 1 (5.9) | 193 (26.6) |
| ≥75 |  | 3,645 (36.0) | 253 (42.0) | 66 (30.1) | 322 (33.7) | 115 (38.2) | 129 (14.8) | 65 (21.8) | 4,595 (34.4) |  | 61 (12.8) | 3 (13.0) | 0 | 1 (6.7) | 2 (22.2) | 1 (0.6) | 0 | 68 (9.4) |
| Comorbidities (%) |  |  |  |  |  |  |  |  |  |  |  |  |  |  |  |  |  |  |
| Hypertension |  | 6770 (67.0) | 395 (65.6) | 151 (68.9) | 595 (62.2) | 198 (65.8) | 580 (66.5) | 176 (59.1) | 8865 (66.4) |  | 301 (63.0) | 13 (56.5) | 11 (61.1) | 10 (66.7) | 5 (55.6) | 84 (50.9) | 11 (64.7) | 435 (60.0) |
| Ischaemic  heart disease |  | 2703 (26.7) | 176 (29.2) | 47 (21.5) | 265 (27.7) | 80 (26.6) | 271 (31.1) | 55 (18.5) | 3597 (26.9) |  | 82 (17.2) | 2 (8.7) | 2 (11.1) | 4 (26.7) | 1 (11.1) | 19 (11.5) | 1 (5.9) | 111 (15.3) |
| Myocardial  infarction |  | 716 (7.1) | 39 (6.5) | 8 (3.7) | 55 (5.8) | 17 (5.6) | 93 (10.7) | 16 (5.4) | 944 (7.1) |  | 16 (3.3) | 1 (4.3) | 2 (11.1) | 0 | 0 | 4 (2.4) | 0 | 23 (3.2) |
| Heart failure |  | 2327 (23.0) | 113 (18.8) | 27 (12.3) | 172 (18.0) | 82 (27.2) | 244 (28.0) | 60 (20.1) | 3025 (22.6) |  | 49 (10.3) | 1 (4.3) | 3 (16.7) | 1 (6.7) | 1 (11.1) | 10 (6.1) | 1 (5.9) | 66 (9.1) |
| Stroke |  | 2115 (20.9) | 120 (19.9) | 49 (22.4) | 165 (17.3) | 48 (15.9) | 118 (13.5) | 52 (17.4) | 2667 (20.0) |  | 83 (17.4) | 2 (8.7) | 2 (11.1) | 2 (13.3) | 2 (22.2) | 11 (6.7) | 5 (29.4) | 107 (14.8) |
| Renal  impairment |  | 1916 (18.9) | 59 (9.8) | 30 (13.7) | 194 (20.3) | 73 (24.3) | 107 (12.3) | 81 (27.2) | 2460 (18.4) |  | 38 (7.9) | 4 (17.4) | 3 (16.7) | 2 (13.3) | 1 (11.1) | 30 (18.2) | 3 (17.6) | 81 (11.2) |
| Diabetic foot |  | 269 (2.7) | 19 (3.2) | 6 (2.7) | 27 (2.8) | 12 (4.0) | 17 (1.9) | 13 (4.4) | 363 (2.7) |  | 6 (1.3) | 0 | 1 (5.6) | 1 (6.7) | 0 | 1 (0.6) | 1 (5.9) | 10 (1.4) |
| Hepatic  impairment  - Severe |  | 530 (5.2) | 42 (7.0) | 14 (6.4) | 49 (5.1) | 15 (5.0) | 21 (2.4) | 10 (3.4) | 681 (5.1) |  | 12 (2.5) | 1 (4.3) | 3 (16.7) | 0 | 1 (11.1) | 1 (0.6) | 1 (5.9) | 19 (2.6) |
| Lactic  acidosis |  | 1 (0.0) | 0 | 0 | 1 (0.1) | 0 | 0 | 0 | 2 (0.0) |  | 0 | 0 | 0 | 0 | 0 | 0 | 0 | 0 |
| T1DM |  | 96 (0.9) | 6 (1.0) | 1 (0.5) | 80 (8.4) | 5 (1.7) | 61 (7.0) | 3 (1.0) | 252 (1.9) |  | 3 (0.6) | 0 | 0 | 0 | 0 | 4 (2.4) | 1 (5.9) | 8 (1.1) |

Data are presented as mean (standard deviation), or n (%).

Table S4 Descriptive summary of observed HbA1c value at 4 months with unadjusted population who had HbA1c value at 4 months

| Cohort |  | Monotherapy cohort at index date-1 (N=10,346) | | | | | | |  | Combination therapy cohort at index date-2 (N=851) | | | | | | |
| --- | --- | --- | --- | --- | --- | --- | --- | --- | --- | --- | --- | --- | --- | --- | --- | --- |
| Drug class |  | DPP-4i | SU | TZD | α-GI | Glinide | SGLT2i | GLP-1Ra |  | DPP-4i | SU | TZD | α-GI | Glinide | SGLT2i | GLP-1Ra |
| n |  | 7,856 | 424 | 156 | 747 | 236 | 676 | 251 |  | 554 | 41 | 25 | 23 | 14 | 173 | 21 |
| HbA1c (%) |  | 6.7 (0.9) | 6.9 (1.1) | 6.7 (1.0) | 6.8 (1.1) | 6.6 (0.9) | 7.0 (1.0) | 6.7 (1.1) |  | 7.1 (1.0) | 7.4 (1.4) | 6.9 (0.9) | 7.0 (0.9) | 6.8 (0.8) | 7.2 (1.0) | 6.9 (1.1) |
| HbA1c (mmol/mol) |  | 50 (9) | 52 (12) | 50 (11) | 51 (12) | 49 (10) | 52 (11) | 50 (12) |  | 54 (11) | 57 (15) | 52 (10) | 52 (10) | 50 (9) | 56 (11) | 52 (12) |

Data are presented as mean (standard deviation).

a)


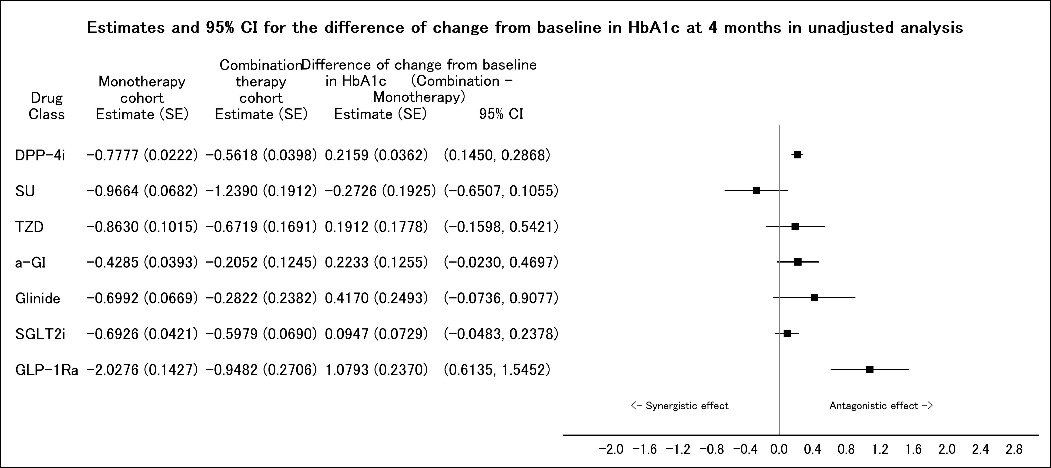


b)


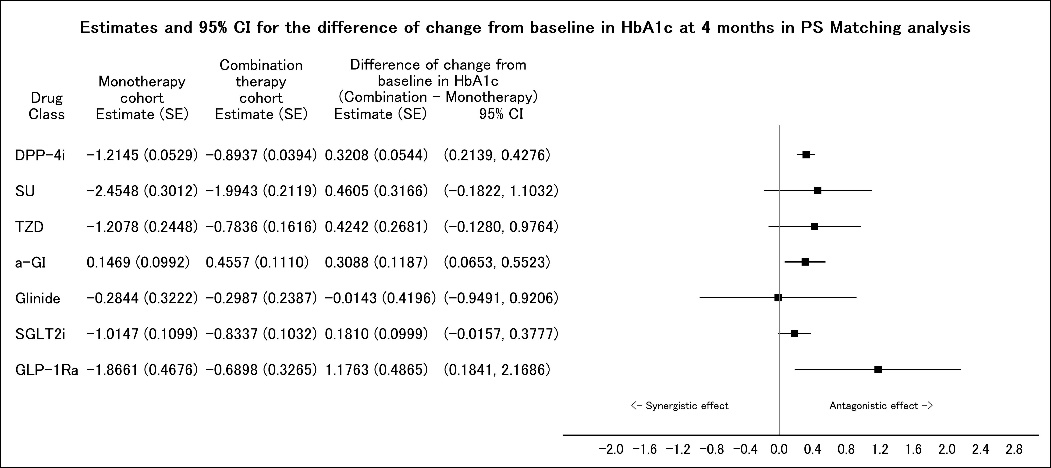


c)


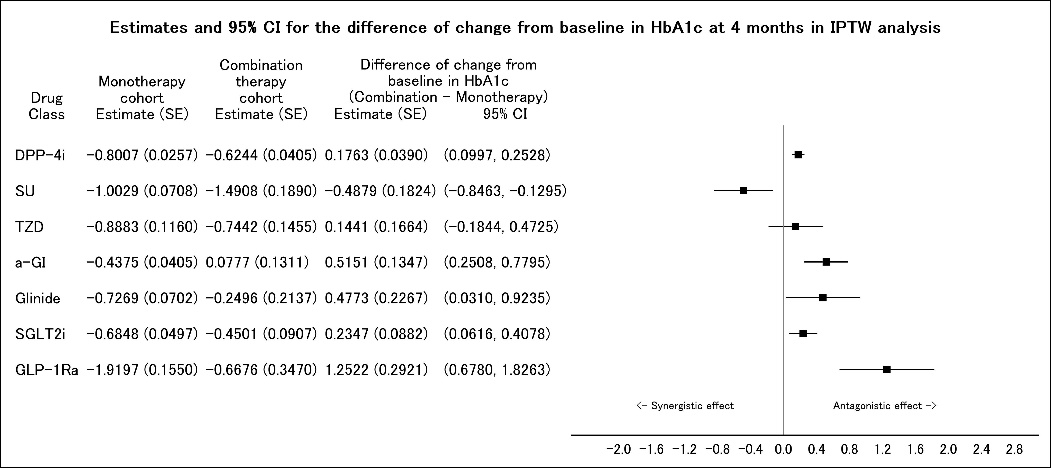


Figure S1 Plot of MMRM for difference of change from baseline in HbA1c at 4 months between cohorts with unadjusted analysis (a), PSM-adjusted analysis (b) and IPTW-adjusted analysis (c): Sensitivity analysis for patients with ≥90 days between index date-1 and index date-2 in combination therapy cohort
